# Linking Systemic Inflammation to Coronary Lesion Complexity: A Combined FFR and OCT Study

**DOI:** 10.3390/ijms262110683

**Published:** 2025-11-02

**Authors:** Nicoleta-Monica Popa-Fotea, Miruna-Mihaela Micheu, Lucian Calmac, Alina Scarlatescu, Diana Zamfir, Cosmin Mihai, Vlad Bataila, Bogdan Marian Drăgoescu, Vlad Ploscaru, Radu Popescu, Raluca-Elena Mitran, Ana-Maria Bacaliaro, Daniel Tonu, Alexandru Scafa-Udriște

**Affiliations:** 1 Department of Cardiology, University of Medicine and Pharmacy Carol Davila, 8, Eroii Sanitari, 050474 Bucharest, Romania; fotea.nicoleta@yahoo.com (N.-M.P.-F.); lcalmac@gmail.com (L.C.); alina.scarlatescu@gmail.com (A.S.); diana_zam74@yahoo.com (D.Z.); cosmihalp@yahoo.com (C.M.); vladbataila@yahoo.co.uk (V.B.); marian-bogdan.dragoescu@drd.umfcd.ro (B.M.D.); vlad_ploscaru86@yahoo.com (V.P.); radu.popescu300397@gmail.com (R.P.); anamariamihail94@gmail.com (A.-M.B.); danieltonu1994@gmail.com (D.T.); alexandru.scafa@umfcd.ro (A.S.-U.); 2 Emergency Clinical Hospital, 8, Calea Floreasca, 014461 Bucharest, Romania; ralucamitran972@gmail.com

**Keywords:** interleukin-1 receptor antagonist, resistin, C-reactive protein, fractional flow reserve, optical coherence tomography

## Abstract

Residual inflammatory risk after acute coronary syndromes (ACSs) remains a critical contributor to atherosclerosis progression and plaque destabilization. Inflammatory biomarkers such as interleukin-1 receptor antagonist (IL-1ra), resistin, and C-reactive protein (CRP) may provide additional insights into coronary lesion complexity and vulnerability. The main aim of the study was to evaluate the association of interleukin-1 receptor antagonist (IL-1ra), resistin, and C-reactive protein (CRP) with coronary disease extent; functional significance of non-culprit lesions, assessed by fractional flow reserve (FFR); and plaque vulnerability, assessed by optical coherence tomography (OCT) in patients with acute coronary syndrome (ACS). This prospective study enrolled 93 ACS patients undergoing invasive coronary assessment for an ACS. Inflammatory biomarkers were measured at admission and 6 months post-event. Patients were stratified post hoc into tertiles by biomarker distribution. SYNTAX score, FFR, and OCT-defined thin-cap fibroatheroma (TCFA) were used to characterize lesion burden and morphology. Multivariate logistic regression was performed adjusting for conventional cardiovascular risk factors and ACS type. Higher tertiles of IL-1ra, resistin, and CRP were significantly associated with increased SYNTAX score (*p* < 0.05), FFR < 0.80 (68% in the highest tertile), and presence of TCFA (62% vs. 20%, *p* < 0.01). All biomarkers correlated with coronary disease severity. In multivariate logistic models, IL-1ra (OR 1.23 per 100 pg/mL, *p* = 0.03), resistin (OR 2.35 per 1 ng/mL, *p* = 0.001), and CRP (OR 1.11 per 0.001 ng/mL, *p* = 0.006) independently predicted high-risk coronary profiles. IL-1ra, resistin, and CRP are independently associated with lesion complexity, functional significance, and vulnerability in ACS. Inflammatory biomarker profiling may provide complementary anatomical and physiological assessment in future ACS risk stratification strategies.

## 1. Introduction

Despite the vast improvements in the prevention, diagnosis, and treatment of coronary artery disease (CAD), it remains one of the leading causes of morbidity and mortality worldwide [1]. Inflammation plays a central role in the pathophysiology of CAD linked with the development of atherosclerotic plaques. Targeting various inflammatory pathways proved important benefits in improving the prognosis of patients with CAD. Emerging evidence suggests that inflammatory biomarkers such as interleukin-1 receptor antagonist (IL-1ra), resistin, and C-reactive protein (CRP) may reflect both systemic inflammation and local plaque vulnerability. While large-scale studies have established the association between inflammatory biomarkers—particularly CRP—and long-term major adverse cardiovascular events (MACEs), the intermediate pathophysiological steps linking systemic inflammation to residual lesion significance and plaque instability remain less well defined. This study addresses this critical gap by integrating fractional flow reserve (FFR) and optical coherence tomography (OCT), two complementary and clinically validated tools, to characterize the functional and morphological impact of inflammation on non-culprit coronary lesions following ACS. Unlike traditional studies that focus on clinical outcomes, our approach provides mechanistic insight into how systemic inflammation translates into functional ischemia and morphological vulnerability.

The primary objective of the study is to assess the association between IL-1ra, resistin, and CRP levels and coronary lesion complexity (SYNTAX score), functional lesion severity (FFR), and vulnerable plaque features (OCT), while the second objective is to evaluate whether these biomarkers are independent predictors of functionally significant residual lesions (FFR < 0.80) and high-risk plaque morphology thin-cap fibroatheromas (TCFA) < 65 μm.

## 2. Results

The baseline characteristics of the study population, stratified by tertiles of inflammatory biomarker levels (IL-1ra, resistin, and CRP), are summarized in Table 1. Each tertile included 31 patients. With 93 included subjects and the observed event rates typical for our cohort (50–60% for FFR < 0.80; 35–45% for TCFA), the study provides roughly 80% power to detect per-tertile odds ratios ≳ 1.8–2.0 at α = 0.05, which aligns with the effect sizes observed.

There were no statistically significant differences in age across tertiles, with mean values of 56.4 ± 10.5 years in the low inflammation group, 58.1 ± 11.2 years in the intermediate group, and 59.3 ± 10.6 years in the high inflammation group (*p* = 0.429). The proportion of male patients was similar across groups, ranging from 84.2% in the lowest tertile to 90.3% in the highest (*p* = 0.575). Cardiovascular risk factors were evenly distributed among tertiles. The prevalence of smoking was lower in the lowest tertile (50%) compared with the highest tertile (58.1%), but this difference was not statistically significant (*p* = 0.398). Similarly, the prevalence of hypertension was higher across tertiles (68.4% to 78.1%, *p* = 0.339), and dyslipidemia was more common in the highest tertile (81.8%) compared to the lowest (63.2%), though this trend did not reach statistical significance (*p* = 0.092). No significant differences were observed for diabetes mellitus (*p* = 0.813) or prior history of CAD (*p* = 0.386).

Echocardiographic parameters were analyzed according to tertiles of inflammatory biomarker levels. Left ventricular volumes (LVEDV and LVESV), LVEF, global longitudinal strain (GLS), Tei index, and wall motion index (WMI) showed no statistically significant differences across tertiles. However, the E/e′ ratio, reflecting left ventricular filling pressures, was significantly higher in the high inflammation tertile compared to the low tertile (9.01 ± 3.67 vs. 7.54 ± 2.42, *p* = 0.050), suggesting impaired diastolic function in patients with elevated inflammatory burden (Table 2).

### 2.1. Inflammatory Biomarkers and Coronary Disease Extent

IL-1ra levels increased progressively from 502.1 ± 48.3 pg/mL in T1 to 772.5 ± 96.7 pg/mL in T3 (*p* = 0.015). Similarly, resistin levels rose from 9.04 ± 0.50 ng/mL in the low tertile to 18.8 ± 3.2 ng/mL in the high tertile (*p* = 0.005). CRP levels showed the same upward trend, with values of 4201 ± 764 ng/mL in T1 and 8775.7 ± 1048 ng/mL in T3 (*p* = 0.001) (Table 3).

Patients with elevated IL-1ra and resistin levels demonstrated significantly higher SYNTAX scores (median 22.8 vs. 16.3, *p* = 0.03) and a greater number of residual lesions (*p* < 0.01). Correlation analysis showed that all three biomarkers positively correlated with the SYNTAX score (IL-1RA: r = 0.38, *p* = 0.004; resistin: r = 0.41, *p* = 0.002; hs-CRP: r = 0.46, *p* < 0.001), highlighting their association with the angiographic severity of CAD.

In the control group (n = 30, age- and sex-matched subjects without cardiovascular risk factors or coronary calcification), mean plasma levels of IL-1ra, resistin, and CRP were 185 ± 45 pg/mL, 4.1 ± 1.2 ng/mL, and 0.0012 ± 0.0006 ng/mL, respectively. All biomarker levels were significantly lower compared with the ACS cohort (*p* < 0.001 for each comparison), confirming the expected inflammatory activation associated with acute coronary syndromes.

### 2.2. Association Between Inflammatory Biomarker Tertiles and Functional and Morphological Coronary Outcomes

**Figure 1:**
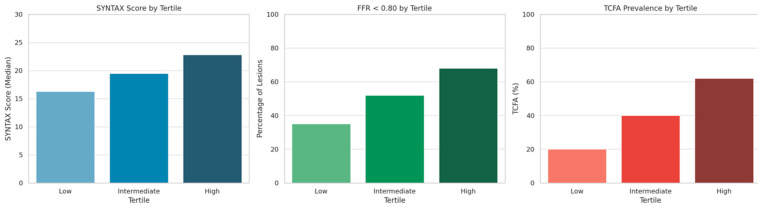
Syntax score, prevalence of functionally significant lesions, and TCFA across inflammatory tertiles.

A progressive increase in the prevalence of functionally significant lesions was observed across tertiles of inflammatory biomarker levels. In patients within the lowest tertile (T1), 35% of lesions demonstrated FFR < 0.80, compared to 52% in the intermediate tertile (T2) and 68% in the highest tertile (T3). This trend supports a strong association between systemic inflammation and the likelihood of ischemia-inducing coronary lesions. Similarly, the presence of TCFA, as assessed by OCT, increased markedly across tertiles. TCFA was identified in 20% of patients in the lowest tertile, rising to 40% in the intermediate tertile, and reaching 62% in the highest tertile. As shown in Figure 1, all three parameters—SYNTAX score, prevalence of FFR < 0.80 lesions, and frequency of TCFA—increased progressively across inflammatory tertiles. These differences were statistically significant (SYNTAX score, *p* = 0.03; FFR < 0.80: *p* = 0.02; TCFA presence: *p* < 0.01).

### 2.3. Multivariate Logistic Regression

After adjusting for conventional cardiovascular risk factors, all three inflammatory biomarkers-IL-1ra, resistin, and CRP-were independently associated with the presence of a high-risk coronary profile, defined as either functionally significant lesions (FFR < 0.80) or TFCA. While the composite inflammatory z-score confirmed a stepwise increase in SYNTAX score, prevalence of FFR < 0.80, and TCFA across tertiles (all *p* < 0.01), multivariate models using individual biomarkers showed differing strengths of association-resistin (OR 2.35 per 1 ng/mL, *p* = 0.001), CRP (OR 1.11 per 0.001 ng/mL, *p* = 0.006), and IL-1ra (OR 1.23 per 100 pg/mL, *p* = 0.03)-underscoring that each marker captures a distinct inflammatory pathway (Table 4).

### 2.4. Inflammatory Biomarkers at 6-Month Follow-Up

At 6-month follow-up, levels of IL-1ra, resistin, and CRP remained higher in patients within the upper tertile of inflammatory burden, although the differences between tertiles were no longer statistically significant. IL-1ra levels showed a numerical increase from 287.2 ± 16.4 pg/mL in the lowest tertile to 382.0 ± 25.0 pg/mL in the highest (*p*-for-trend = 0.051). A similar pattern was observed for CRP (3211.1 ± 780.3 vs. 5262.9 ± 845.8 ng/mL, *p*-for-trend = 0.062) and resistin (6.48 ± 2.72 vs. 7.69 ± 5.38 ng/mL, *p*-for-trend = 0.49). Table 5 presents the comparison between the lowest (T1) and highest (T3) inflammatory tertiles, representing the extreme contrast in systemic inflammation. These findings suggest a trend toward persistent low-grade inflammation in a subset of patients, potentially contributing to long-term cardiovascular risk, even after clinical stabilization post-ACS.

## 3. Discussion

C-reactive protein remains one of the most studied acute-phase proteins in ischemic heart disease. However, CRP is not helpful for the diagnosis and treatment of acute coronary syndrome patients, but its measurement after ACS and PCI identifies those patients at risk for further complications and worse prognosis [2]. Ridker PM et al. have shown that high-sensitivity CRP is a stronger predictor of future major adverse cardiovascular events (MACEs) than low-density lipoprotein cholesterol in patients with or at high risk of atherosclerotic disease [3]. In addition to CRP, which is a well-established marker of systemic inflammation and cardiovascular risk, we selected IL-1ra and resistin based on their distinct and complementary roles in the inflammatory cascade and atherosclerotic pathobiology.

IL-1ra is a naturally occurring anti-inflammatory cytokine that modulates the activity of IL-1, a key upstream mediator in atherogenesis, endothelial dysfunction, and plaque destabilization. Elevated IL-1ra levels have been associated with adverse outcomes in acute coronary syndromes and reflect an endogenous counter-regulatory response to inflammation. Its relevance is further supported by the mechanistic and clinical data from trials targeting the IL-1 axis (e.g., CANTOS [4], VCUART/VCUART2 [5], and VCUART3 [6]).

Resistin, originally identified in adipocytes, is now recognized as a pro-inflammatory cytokine predominantly secreted by mononuclear cells in humans. It promotes endothelial activation, smooth muscle proliferation, and foam cell formation and has been independently linked to a subset population, precisely those with type 2 diabetes mellitus [7]. Since 2005 it has been proved to be an independent inflammatory marker of atherosclerosis [8]. Although the association of resistin with CAD and its severity was shown in different cohorts (NSTEMI, STEMI, stable angina) [9,10,11], the impact of this cytokine on the expanse of coronary lesions in subjects with ACS displaying other lesions apart from the culprit evaluated with extensive functional and anatomical tests such as FFR and OCT was not previously evaluated.

Unlike CRP, which is a downstream acute-phase reactant, IL-1ra and resistin capture distinct aspects of early immune activation and cytokine-mediated plaque remodeling, making them attractive candidates for exploring the inflammatory phenotype of coronary lesions using intracoronary imaging. The study of one single cytokine role in atherosclerosis is extremely difficult, seeing that some of these biomarkers induce the activity of others; for example, IL-1 induces IL-6, IL-18, and CRP [12], for which a combination of cytokines is possibly a better choice. At present CRP remains the preferred inflammatory biomarker for cardiovascular risk stratification. The use of multiple markers gives a better picture of the pro-inflammatory cascade, although the majority of studies investigated single cytokines. Beyond CRP, both IL-1ra and resistin exhibit specific temporal patterns during acute myocardial infarction. IL-1ra typically increases within the first 24 h following myocardial injury, reflecting a compensatory anti-inflammatory response to IL-1β activation, and may remain elevated for several days, as shown in the VCUART trials [5,6]. In contrast, resistin, primarily released by activated monocytes, peaks slightly later (24–48 h) and mirrors ongoing monocyte/macrophage activation and endothelial dysfunction rather than acute tissue necrosis. In our study, blood sampling performed 2–5 days post-admission likely captured both phases—the peak of the IL-1ra response and the early plateau of resistin—thus representing a stable inflammatory state rather than the immediate periprocedural fluctuation.

The present findings from our study align with and extend previous reports on the inflammatory mechanisms driving coronary instability. Using a combination of FFR and OCT, we identified a strong relationship between systemic inflammation and high-risk coronary anatomy, beyond traditional cardiovascular risk factors. Multiple studies, including the AtheroGene [13], PROVE-IT TIMI-22 [14], and CANTOS [4] trials, have emphasized the prognostic importance of persistent systemic inflammation despite optimal lipid lowering. Elevated CRP levels reflect activation of the IL-1/IL-6 axis and are strongly linked to plaque vulnerability, myocardial necrosis, and recurrent ischemia. The LoDoCo2 [15] and COLCOT [16] studies further supported the prognostic relevance of persistent CRP elevation, showing that anti-inflammatory therapies such as low-dose colchicine improved outcomes in stable and post-ACS patients. Although the per-unit odd ratio for CRP (1.11) represents a modest effect size, its clinical implication becomes evident when extrapolated across the range of CRP values typically observed in ACS. A 0.005 ng/mL increase in CRP corresponds to nearly 1.7-fold higher odds of anatomically complex and functionally significant coronary lesions.

Similarly, increased resistin and IL-1ra levels have been associated with macrophage activation and impaired endothelial repair. Our observation that these biomarkers correlate with both functional (FFR) and morphological (OCT-defined TCFA) indices of coronary vulnerability supports the concept that inflammation and microstructural plaque instability are tightly interconnected. Comparable trends were observed in the VCU-ART studies [5,6], where IL-1 blockade reduced ventricular remodeling after MI, and in observational cohorts linking resistin to adverse cardiac remodeling and mortality.

Taken together, these findings reinforce the pathophysiological continuum connecting systemic inflammation, coronary plaque destabilization, and ischemia-driven events.

Our findings are aligned with this evidence, indicating that even in a population uniformly treated with high-intensity statins, elevated IL-1ra and CRP levels are independently associated with greater lesion complexity, ischemia-inducing stenoses, and plaque vulnerability. To our knowledge, this is the first analysis correlating these biomarkers with the anatomical and physiological complexity of non-culprit coronary lesions, offering a novel integrative approach to residual inflammatory risk evaluation.

### 3.1. Inflammation and Coronary Disease Burden

Our results show that patients in the highest tertile of inflammatory markers had significantly higher SYNTAX scores, more residual lesions, and a greater likelihood of multivessel disease. This reinforces the established paradigm that inflammation is not only central to the initiation of atherosclerosis but also contributes to its diffuse nature and progression. Notably, IL-1ra, CRP, and resistin levels were closely correlated with coronary lesion burden, underscoring the relevance of these mediators in amplifying vascular injury and plaque complexity.

### 3.2. Functional Relevance of Inflammation

A key strength of our study is the incorporation of FFR in the assessment of non-culprit lesions. Patients with high resistin, CRP, and IL-1RA levels had a significantly greater proportion of FFR-positive lesions (FFR < 0.80), suggesting that inflammatory burden is linked to the functional significance of residual stenoses. This highlights the value of combining anatomical and physiological assessments in understanding the full clinical impact of coronary disease.

### 3.3. Plaque Vulnerability and OCT Insights

Beyond angiographic and functional characterization, we employed OCT to directly assess the presence of TFA. High levels of resistin, CRP, and IL-1ra were associated with TCFA and thus more likelihood of plaque instability.

### 3.4. Multivariate Predictive Value

In multivariate logistic regression, all three biomarkers independently predicted a high-risk coronary profile, defined as either FFR < 0.80 or TFCA. Resistin and CRP demonstrated the strongest predictive power, while IL-1ra also emerged as a significant marker after adjustment, reinforcing the value of a multi-biomarker approach in identifying high-risk patients. Adjustment variables were selected based on biological plausibility and prior evidence rather than statistical association. ACS type was excluded from the final model to avoid potential collider bias, as it may lie on the causal pathway between systemic inflammation and plaque instability.

## 4. Material and Methods

### 4.1. Study Population

The present study was observational, prospective, and single-center, conducted at the Emergency Clinical Hospital, Bucharest, between 2020 and 2023. All patients were included only after signing an informed consent according to the guidelines of the Declaration of Helsinki. The study was approved by the Ethics Committee of Clinical Emergency Hospital Bucharest, approval number 9013/28.09.2018. All subsequent subjects with ACS were referred for the study, their eligibility being checked following the inclusion and exclusion criteria (Supplementary Figure S1). The inclusion criteria were acute coronary syndrome in the first 5 days after the acute event with at least another coronary lesion apart from the culprit one estimated to have a diameter stenosis ≥ 40% on coronary angiography in subjects with a life expectancy of at least one year. The exclusion criteria were cardiogenic shock, surgical revascularization indication, hepatitis virus B or C or human immunodeficiency virus, ACS with onset more than 5 days earlier or another ACS during the last 6 months, large surgical interventions in the last three months, and chronic renal failure with a glomerular filtration rate < 30 mL/min/1.73 m^2^. We also excluded from our cohort patients with any acute or chronic inflammatory, autoimmune, or infectious disease (including rheumatoid arthritis, systemic lupus erythematosus, vasculitis, chronic obstructive pulmonary disease with recent exacerbation, or active infection), as well as those with known malignancy or chronic liver disease, to avoid potential confounding effects on systemic inflammatory biomarkers. For the extensive protocol of the study, see the previous publication [13]. ACS type was categorized according to standard ESC 2020 diagnostic criteria into ST-segment elevation myocardial infarction (STEMI) and non–ST-segment elevation acute coronary syndromes (NSTEMI/UA) [17]. Given the small number of unstable angina cases, NSTEMI and UA were analyzed together for statistical purposes.

A separate age- and sex-matched control group (30 subjects) was included to provide internally consistent baseline biomarker values obtained under identical analytical conditions, thereby avoiding potential bias from external population-based reference ranges. Controls had no history of cardiovascular disease or traditional risk factors (hypertension, dyslipidemia, diabetes mellitus, or obesity) and had a calcium score of zero on coronary CT angiography. These individuals were not subjected to FFR, OCT, or angiography and were used solely for biomarker baseline comparisons.

### 4.2. Blood Sample Harvesting

The blood probes were harvested by peripheral venous puncture at 2–5 days after admission for ACS (named T1) and at 6 months after the index event (named T2). Blood was harvested in EDTA tubes and worked within 30 min after harvesting. Plasma was obtained by 15 min centrifugation at 1000 g. Afterwards, plasma was aliquoted and stored at <−20 °C until processed. For the control group, blood was harvested at enrollment.

### 4.3. The Inflammatory Cytokines Assay Procedure

Enzyme-linked immunosorbent assay (ELISA) was the method used to quantitatively determine hs-CRP, resistin, and IL-1ra. R&D Human System kits were followed for each cytokine (Human CRP Quantikine ELISA Kit, USA R&D Systems, Inc., Minneapolis, USA [18], Human IL-1ra/IL-1F3 Quantikine ELISA [19], USA R&D Systems, Inc., Minneapolis, USA and Human Resistin ELISA Kit [20], R&D Systems Inc., Minneapolis, MN, USA). C-reactive protein intra-assay coefficient of variation (CV) was between 3.6 and 4.6%, while inter-assay CV was between 5.6 and 7%. The minimum detectable dose (MDD) was 0.011–0.037 ng/mL (mean MDD was 0.022 ng/mL). The human IL-1ra had an intra-assay CV between 3.7 and 7% and an inter-assay CV of 6.7–11%, with the minimum detectable dose (MDD) ranging from 2.2 to 18.3 pg/mL (mean MDD 6.3 pg/mL). The human resistin intra-assay precision was between 3.8 and 5.3%, and the inter-assay CV was between 7.8 and 9.2%, with an MDD range from 0.010 to 0.055 ng/mL (mean MDD 0.026 ng/mL). Based on the distribution of inflammatory biomarker levels (IL-1ra, resistin, and CRP), patients were stratified post-hoc into tertiles of low (T1), intermediate (T2), and high (T3) inflammatory burden.

### 4.4. Transthoracic Echocardiography

Echocardiography parameters were measured using a GE Healthcare Vivid E95 machine, making use of a 4–12 MHz transthoracic probe (GE 12S-D) for 2D and a 4–10 MHz transthoracic probe for 3D data acquisitions (GE 4VC-D). All acquired images were analyzed offline on an Echo Pac version v206 workstation by two different expert investigators at least two times to reduce inter- and intra-variability biases. Standard apical views (four- and two-chamber, apical long-axis view) and parasternal long and short axes were acquired with 50–70 frames per second. Conventional echocardiographic measurements were accomplished in accordance with current guidelines [17]. Left ventricle GLS was calculated as the average of the 17 LV segments using speckle tracking echography. For a better functional assessment of the left ventricle, 3D echocardiography was performed: measurement of LVESV, LVEDV, and 3D LVEF. All measurements were performed at baseline (between 2 and 5 days after ACS) and at 6 months follow-up.

### 4.5. Coronary Angiography

All patients included in this study underwent coronary angiography at admission, in accordance with current ACS management guidelines. Patients who had at least one additional coronary lesion apart from the culprit, with a diameter stenosis ≥ 40% on angiography, were enrolled in the study. Fractional flow reserve (FFR) and optical coherence tomography (OCT) assessments were performed exclusively on non-culprit lesions exhibiting ≥ 40% diameter stenosis by angiography. The culprit lesions responsible for the acute coronary event were not evaluated using FFR or OCT because of ethical and procedural considerations in the context of acute revascularization. The SYNTAX score was used to grade the complexity of coronary artery disease (CAD) by two independent interventional cardiologists. In the OCT analysis of non-culprit lesions, thin-cap fibroatheromas (TCFA), defined as plaques with a fibrous cap thickness < 65 μm, were specifically identified and recorded. All imaging evaluations (FFR, OCT, and echocardiography) were performed by experienced operators who were blinded to biomarker results. For OCT and FFR measurements, intraobserver and interobserver variability were assessed in a random subset of 20 patients, showing excellent reproducibility (intraobserver ICC = 0.94; interobserver ICC = 0.91).

### 4.6. Statistics

The analysis was conducted using the SPSS statistical software program version 21 (IBM, Armonk, NY, USA). Values of *p* were two-tailed, and values < 0.05 were considered statistically significant. Data were presented as mean ± SD for continuous variables and as number and percentage for categorical variables. For the sample size calculation, considering that the primary mechanistic question was the association between inflammatory burden and coronary disease complexity, assuming the detection of a modest correlation (r ≈ 0.30) between biomarker levels and SYNTAX score at α = 0.05 and 80% power, the required sample was around 84.

To reflect overall inflammatory burden, we generated a composite inflammatory score by standardizing each biomarker value (IL-1ra, resistin, and CRP) into z-scores and calculating their sum for each patient. This composite score integrates multiple inflammatory pathways and provides a more comprehensive profile of systemic inflammation. Patients were then stratified post-hoc into tertiles based on the distribution of this composite score, corresponding to low (T1), intermediate (T2), and high (T3) inflammatory status, based on the distribution of each biomarker: IL-1ra: T1 < 560 pg/mL, T2 = 560–720 pg/mL, T3 > 720 pg/mL; resistin: T1 < 11.0 ng/mL, T2 = 11.0–16.0 ng/mL, T3 > 16.0 ng/mL; CRP: T1 < 0.005 ng/mL, T2 = 0.005–0.008 ng/mL, T3 > 0.008 ng/mL. In addition, a composite inflammatory score was generated by converting each biomarker to a standardized z-value and summing the three z-scores for each participant, representing overall inflammatory burden. Comparisons across tertiles were complemented by regression-based tests for trend. For continuous variables, linear regression models with tertiles entered as an ordinal variable were used to estimate *p*-for-trend, while for categorical outcomes, logistic regression was applied. The *p*-trend values confirmed consistent and significant gradients across increasing levels of inflammatory burden. Correlations between biomarker levels and echocardiographic, angiographic, or morphological parameters were assessed using Spearman’s rank or Pearson’s correlation coefficients, as appropriate. Univariate logistic regression was used to identify potential predictors of adverse coronary or ventricular outcomes. Variables with statistical significance in univariate analyses were entered into multivariate logistic and linear regression models, adjusting for confounding factors including age, sex, diabetes mellitus, and arterial hypertension.

## 5. Conclusions

### 5.1. Clinical Implications

The novelty of our findings lies in bridging systemic inflammation with surrogate but mechanistically relevant intracoronary phenotypes using FFR and OCT. While biomarkers like CRP are well-known predictors of MACE, their direct link to residual lesion ischemia and plaque instability has been largely unexplored in ACS patients post-revascularization. By demonstrating that elevated inflammatory markers correlate with FFR-positive lesions and TCFA, we offer a potential intermediate endpoint that may be used to refine post-ACS risk stratification and personalize follow-up even before clinical events occur. From the clinical perspective, our results underscore the need to complement lipid-lowering therapy with inflammation-targeted approaches. Trials such as CANTOS (canakinumab), COLCOT, and LoDoCo2 (colchicine) have demonstrated that modulating the inflammatory response reduces recurrent cardiovascular events independently of LDL-C levels. In this context, identifying patients with persistently elevated inflammatory biomarkers-such as CRP, resistin, or IL-1ra-after ACS may help guide adjunctive anti-inflammatory therapies and refine risk stratification beyond conventional metrics.

Furthermore, combining biomarker assessment with high-resolution coronary imaging could help select individuals most likely to benefit from such interventions, providing a precision-medicine framework for secondary prevention.

### 5.2. Limitations

Although we report statistically significant associations between inflammatory biomarkers and both functional and morphological features of coronary disease, our study design does not allow for causal inference. Given the modest sample size and the inclusion of several covariates in multivariate models, the possibility of model overfitting cannot be entirely excluded. These analyses should therefore be interpreted as exploratory and hypothesis-generating, requiring confirmation in larger cohorts. It remains unclear whether systemic inflammation drives plaque progression and functional impairment or whether advanced lesions induce greater inflammatory responses. Longitudinal studies with serial imaging and biomarker measurements are needed to determine the temporal sequence and potential causal pathways underlying these associations. We acknowledge that CRP, IL-1ra, and resistin levels are acutely elevated during the index ACS event and may not reflect baseline chronic inflammation. Our findings therefore characterize the acute inflammatory milieu rather than long-term exposure. Nonetheless, the persistence of elevated inflammatory markers at 6-month follow-up in a subset of patients suggests that, for some individuals, acute elevations may indicate a sustained pro-inflammatory state. Future studies including pre-event or post-stabilization biomarker measurements are warranted to differentiate transient from chronic inflammation.

In patients with acute coronary syndromes, elevated levels of IL-1ra, resistin, and CRP were independently associated with greater lesion complexity, functional significance, and plaque vulnerability. These results should be interpreted within the specific scenario of post-ACS patients evaluated with combined FFR and OCT, and further validation in larger, longitudinal cohorts is warranted before broader extrapolation. Integrating inflammation profiling into post-ACS evaluation protocols may ultimately refine prognosis and guide personalized therapeutic strategies.

## Figures and Tables

**Table 1:** Demographic, cardiovascular risk factors, and other paraclinical parameters by inflammation tertile.

Characteristic	Tertile 1	Tertile 2	Tertile 3	*p*-for-Trend
Age (years)	56.4 ± 10.5	58.1 ± 11.2	59.3 ± 10.6	0.41
Male sex (%)	84.2	86.8	90.3	0.56
Smoking (%)	50.0	52.6	58.1	0.39
Hypertension (%)	68.4	71.1	78.1	0.28
Dyslipidemia (%)	63.2	72.3	81.8	0.07
Diabetes mellitus (%)	26.3	27.6	25.8	0.81
Prior CAD (%)	10.5	11.8	18.1	0.33
Platelet count (10^3^/μL)	296.5 ± 19.0	294.3 ± 20.1	292.1 ± 21.3	0.64
Creatinine (mg/dL)	0.91 ± 0.18	0.90 ± 0.17	0.89 ± 0.18	0.57
Total cholesterol (mg/dL)	223.3 ± 49.6	224.2 ± 50.2	224.9 ± 53.4	0.88
Triglycerides (mg/dL)	214.1 ± 27.7	200.6 ± 138.1	191.8 ± 16.8	0.47

Values are expressed as mean ± SD or frequency (%).

**Table 2:** Echocardiographic parameters by inflammation tertile.

Parameter	Tertile 1	Tertile 2	Tertile 3	*p*-for-Trend
LVEDV 3D (mL)	108.9 ± 24.9	107.0 ± 25.0	106.2 ± 29.4	0.65
LVESV 3D (mL)	53.2 ± 15.5	54.0 ± 18.0	55.2 ± 20.9	0.60
LVEF 3D (%)	50.6 ± 7.1	49.5 ± 7.0	48.8 ± 7.1	0.24
LV GLS (%)	−13.73 ± 3.11	−13.75 ± 3.10	−13.78 ± 3.03	0.93
E/e′ ratio	7.54 ± 2.42	8.30 ± 3.19	9.01 ± 3.67	0.045
Tei index	0.51 ± 0.21	0.51 ± 0.23	0.52 ± 0.25	0.84
Wall Motion Index	1.35 ± 0.21	1.34 ± 0.21	1.33 ± 0.21	0.72

Values are expressed as mean ± SD. GLS: global longitudinal strain; LVEDV: left ventricular end-diastolic volume; LVEF: left ventricular ejection fraction; LVESV: left ventricular end-systolic volume; WMI: wall motion index.

**Table 3:** Inflammatory marker distribution in each tertile.

Parameter	Tertile 1	Tertile 2	Tertile 3	*p*-for-Trend
IL-1RA (pg/mL)	480 ± 40	640 ± 50	770 ± 60	0.012
Resistin (ng/mL)	8.9 ± 0.5	13.5 ± 0.6	18.8 ± 3.2	0.004
CRP (ng/mL)	4100 ± 700	6800 ± 600	8775 ± 1050	<0.001
SYNTAX score	16.3 ± 6.2	19.5 ± 7.0	22.8 ± 8.1	0.028
FFR < 0.80 (%)	35.0	52.0	68.0	0.018
TCFA (%)	20.0	40.0	62.0	0.006

Values are expressed as mean ± SD or frequency (%); *p*-for-trend values were obtained from linear regression with tertiles entered as an ordinal predictor (1–3). CRP C-reactive protein; IL-1RA interleukin-1 receptor antagonist.

**Table 4:** Multivariate logistic regression analysis.

Variable	OR	95% CI	*p*-Value
Age	0.983	0.898–1.076	0.704
IL-1ra (per 100 pg/mL)	1.23	1.02–1.48	0.03 *
Resistin (per 1 ng/mL)	2.347	1.533–3.593	0.001 *
CRP (per 1 ng/mL)	1.11	1–2.548	0.006 *
History of CAD	0.126	0.008–2.034	0.145
Smoking	0.141	0.014–1.377	0.092
Hypertension	0.485	0.132–1.782	0.274
Diabetes	1.504	0.179–12.638	0.707
Dyslipidemia	1.644	0.199–13.590	0.645

CAD coronary artery disease; CI confidence interval; CRP C-reactive protein; IL-1ra interleukin-1 receptor antagonist; OR odd ratio. * indicates *p* < 0.05 compared with tertile 1 (reference group).

**Table 5:** Inflammatory biomarkers by tertile at 6-month follow-up.

Biomarker	Tertile 1	Tertile 2	Tertile 3	*p*-for-Trend
IL-1ra (pg/mL)	287.2 ± 16.4	345.0 ± 22.0	382.0 ± 25.0	0.051
Resistin (ng/mL)	6.48 ± 2.72	7.00 ± 3.00	7.69 ± 5.38	0.49
CRP (ng/mL)	3211.1 ± 780.3	4466.7 ± 497.9	5262.9 ± 845.8	0.062

Values are expressed as mean ± SD; *p*-for-trend values were obtained from linear regression with tertiles entered as an ordinal predictor (1–3). CRP C-reactive protein; IL-1ra interleukin-1 receptor antagonist.

## Data Availability

The data presented in this study are available on request from the corresponding author. The data are not publicly available due to privacy and/or ethical restriction.

## Supplementary Materials

The following supporting information can be downloaded at: https://www.mdpi.com/article/10.3390/ijms262110683/s1.
