# Supplementary material for: Linking Systemic Inflammation to Coronary Lesion Complexity: A Combined FFR and OCT Study
Source: Int J Mol Sci. 2025 Nov 2;26(21):10683. doi: 10.3390/ijms262110683 (PMC12608127; doi:10.3390/ijms262110683)
Supplement: Supplementary file 1 [file ijms-26-10683-s001.zip › ijms-3916402-supplementary.pdf]

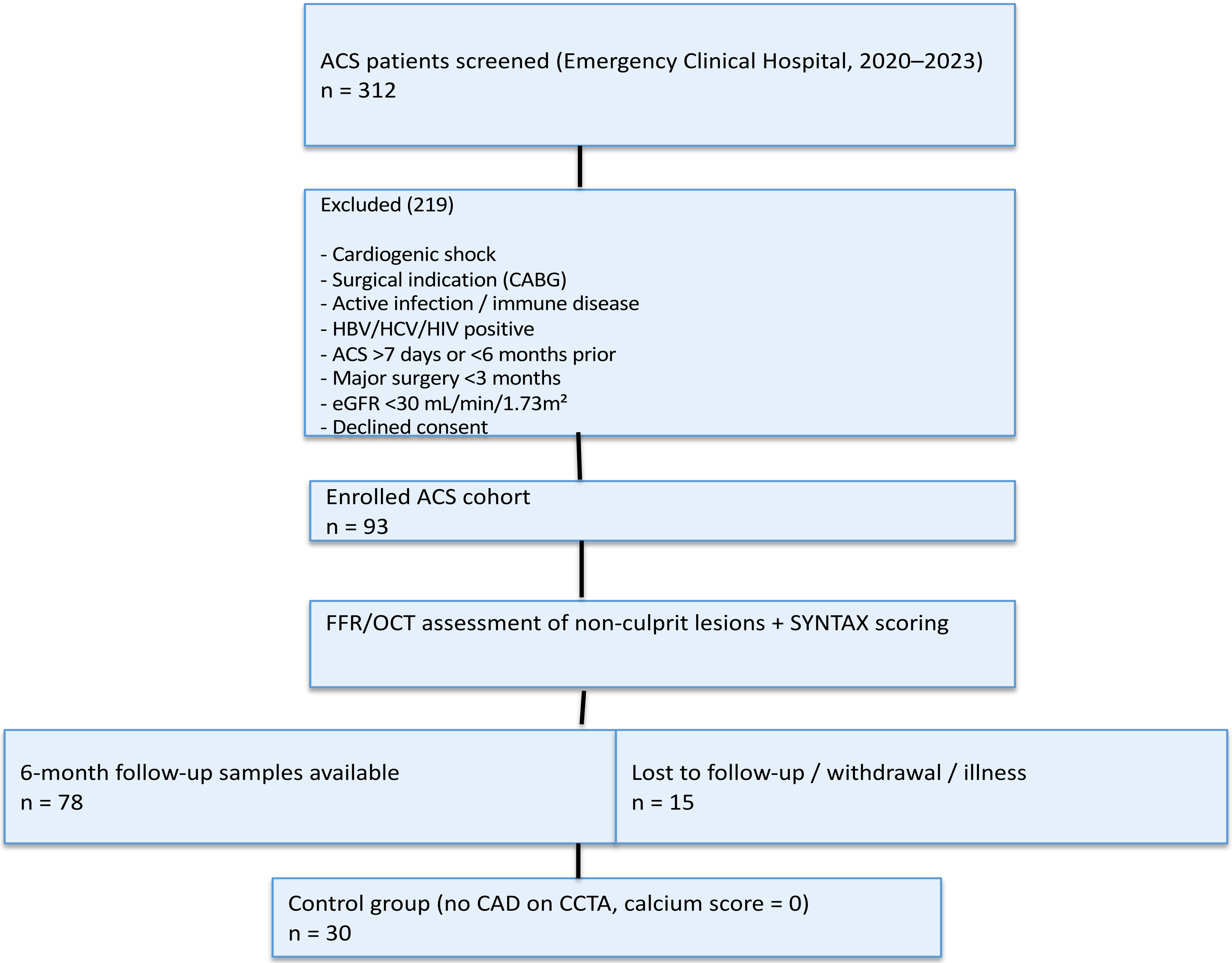
ACS patients screened (Emergency Clinical Hospital, 2020–2023)
n = 312
Excluded (219)
- Cardiogenic shock
- Surgical indication (CABG)
- Active infection / immune disease
- HBV/HCV/HIV positive
- ACS >7 days or <6 months prior
- Major surgery <3 months
- eGFR <30 mL/min/1.73m^2
- Declined consent
Enrolled ACS cohort
n = 93
FFR/OCT assessment of non-culprit lesions + SYNTAX scoring
6-month follow-up samples available
n = 78
Lost to follow-up / withdrawal / illness
n = 15
Control group (no CAD on CCTA, calcium score = 0)
n = 30
